# Editorial: Double-Edged Swords: Genetic Factors That Influence the Pathogenesis of Both Metabolic Disease and Cancer

**DOI:** 10.3389/fendo.2019.00425

**Published:** 2019-07-03

**Authors:** Che-Pei Kung, Maureen E. Murphy, Hua Lu

**Affiliations:** ^1^Washington University School of Medicine, Saint Louis, MO, United States; ^2^Division of Molecular Oncology, Department of Medicine, Siteman Cancer Center, Washington University School of Medicine, Saint Louis, MO, United States; ^3^Program in Molecular and Cellular Oncogenesis, The Wistar Institute, Philadelphia, PA, United States; ^4^Department of Biochemistry and Molecular Biology, Tulane Cancer Center, Tulane University School of Medicine, New Orleans, LA, United States

**Keywords:** cancer, diabetes, metabolic disease, genetic factors, obesity

Our understanding of cancer development has been marked by milestone discoveries in genetics, including the identification and cloning of oncogenes and tumor suppressor genes (1). Successful interventions of cancer, such as hormone therapies of breast cancers and vaccination of HPV, were aided in their development by our knowledge of these critical etiological factors underlying cancer. In contrast, metabolic disease was once thought to be a by-product of modern excessive lifestyles (2). Our increased ability to interrogate massive amounts of genetic information has strengthened the connection between genetics and metabolic disorders. As such, genetics-based therapy for metabolic disease has, at last, become more likely.

In this special research topic, we aim to highlight versatile genetic factors capable of regulating both cancer and metabolic disorders. By examining the existing literature, this collection of review articles provides both comprehensive overview and critical discussion about genetic factors/pathways that are involved in pathogenic mechanisms of multiple diseases, often through under-appreciated aspects of their functions.

The tumor suppressor TP53 is well-known for its role in maintaining genome stability and preventing cancer progression. Recent studies unveiled p53 functions in regulating metabolic homeostasis and diseases, such as diabetes (3). In Zwezdaryk et al. detail our current understanding of how p53 regulates functions of adipose tissues, often paradoxically, to influence the pathogenesis of obesity and cancer. In Gnanapradeepan et al. explain how p53-mediated regulation of gene expression contributes to a novel form of programmed cell death, ferroptosis (iron- and lipid-peroxide-mediated cell death), in the context of metabolic dysfunctions and cancer.

In the nucleus, the stability and transcriptional activity of p53 is promoted by binding to the regulatory region of another tumor suppressor gene, PTEN (4). In Chen et al. elegantly summarize data indicating that, in addition to collaborating with p53, PTEN also regulates tumor metabolism and insulin sensitivity through its phosphatase activity, suggesting PTEN as a potential target for treatment of both cancer and diabetes.

It is now appreciated that cancer cells often repurpose metabolic pathways shared by normal cells to facilitate pro-tumorigenic functions. Goetzman and Prochownik capture this essence with a comprehensive review. In their essay, they thoroughly describe how the oncoprotein c-Myc regulates the metabolic balance in normal, cancerous, and metabolically-defective cell states.

In addition to oncogenes and tumor suppressors, non-coding RNAs also emerge as critical regulators of cellular homeostasis (5). Kong et al. introduce one of the long non-coding RNA (lncRNA), ANRIL. ANRIL resides in a genetic locus that is proximal or overlapping to several cancer-associated genes, including p16-INK4a, ARF (CDKN2a), and p15-INK4b. Potential connections between structural and functional characteristics, as well as unique SNPs (single nucleotide polymorphisms) of ANRIL and their impact on the development of cancer and metabolic diseases such as cardiovascular disease or diabetes are addressed.

Compared to the roles of cancer-associated genes in metabolic disorders, our understanding of how metabolic-disease genes regulate tumorigenesis is only now emerging. Deng et al. focus their mini-review, on one such gene, fat mass and obesity-associated protein (FTO), which acquired its name from epidemiological connections between its SNPs and obesity. Combining the new investigations linking FTO to tumorigenesis and its nature as an mRNA demethylase, development of FTO inhibitors is in full swing to treat both cancer and metabolic disease (6, 7).

Besides individual gene products, classes of genetic factors and pathways that regulate both tumorigenesis and metabolism are also discussed. In Nagarajan et al. summarize our current knowledge about the role of Heparan Sulfate Proteoglycans (HSPGs) in cancer development. Understanding the functions of these “protein-carbohydrate” conjugates, as well as factors regulating their metabolism, offers opportunities to develop treatments against cancers with deregulated HSPGs. Due to the diverse nature of HSPGs, it is not surprising that they are also involved in metabolic disorders like atherosclerosis and obesity (8).

Mechanisms to maintain the balance between biosynthesis and degradation of deoxyribonucleotide triphosphates (dNTPs), the building blocks of DNA, are keys to biological functions. Buj and Aird outline the intensively studied connections between dNTP metabolism and cancer development. They also discuss the recently-identified associations between dNTP homeostasis and metabolic diseases, and the potential novel therapeutic strategies targeting these interconnections for cancer and metabolic disorders.

As the organelle to produce ATP, the mitochondrion is a critical player in maintaining dNTP homeostasis. However, mitochondria also play multiple and important roles in human physiology through a variety of pathways, such as oxidative phosphorylation, production of reactive oxygen species (ROS), and inflammation (9). In Williams and Caino describe how the mitochondrial dynamics (shape and localization) in a cell contributes to cellular homeostasis, and how dysregulation of this network leads to cancer and type II diabetes.

Epigenetic alterations have been implicated in most human diseases, including cancer and metabolic disorders (10). In the review article, Kung et al. conduct a comprehensive review of the literature for the impact of RNA editing enzymes in the development of cancer and metabolic diseases. Importantly, in the era of genome editing, investigations of RNA-editing pathways could lead to promising therapeutic strategies.

To understand the complex nature of human diseases, a great window of opportunity is given by studying versatile players involved in multiple diseases. This collection of well-written reviews offers readers with an updated grand view on the double edged sword roles of genetic factors in connecting cancer with metabolic disorders. For their outstanding works, we sincerely thank all the authors as well as the reviewers/review editors for offering their time and effort for this project, and the editorial team at Frontiers, especially Dr. Emilie Schrepfer, for their helps and professionalism as demonstrated throughout this process.

## Author Contributions

CK drafted the manuscript. CK, MM, and HL reviewed and revised the manuscript.

### Conflict of Interest Statement

The authors declare that the research was conducted in the absence of any commercial or financial relationships that could be construed as a potential conflict of interest.

## References

[1] Lee EY Muller WJ. Oncogenes and tumor suppressor genes. Cold Spring Harb Perspect Biol. (2010) 2:a003236. 10.1101/cshperspect.a003236 20719876 PMC2944361

[2] Gluckman PD Hanson MA. Living with the past: evolution, development, and patterns of disease. Science. (2004) 305:1733–6. 10.1126/science.1095292 15375258

[3] Kung CP Murphy ME. The role of the p53 tumor suppressor in metabolism and diabetes. J Endocrinol. (2016) 231:R61–75. 10.1530/JOE-16-0324 27613337 PMC5148674

[4] Freeman DJ Li AG Wei G Li HH Kertesz N Lesche R. PTEN tumor suppressor regulates p53 protein levels and activity through phosphatase-dependent and -independent mechanisms. Cancer Cell. (2003) 3(2):117–30. 10.1016/S1535-6108(03)00021-7 12620407

[5] Esteller M. Non-coding RNAs in human disease. Nat Rev Genet. (2011) 12:861–74. 10.1038/nrg3074 22094949

[6] Huang Y Su R Sheng Y Dong L Dong Z Xu H. Small-molecule targeting of oncogenic FTO demethylase in acute myeloid leukemia. Cancer Cell. (2019) 35:677–91 e10. 10.1016/j.ccell.2019.03.006 30991027 PMC6812656

[7] Peng S Xiao W Ju D Sun B Hou N Liu Q. Identification of entacapone as a chemical inhibitor of FTO mediating metabolic regulation through FOXO1. Sci Transl Med. (2019) 11:488. 10.1126/scitranslmed.aau7116 30996080

[8] Gordts P Foley EM Lawrence R Sinha R Lameda-Diaz C Deng L. Reducing macrophage proteoglycan sulfation increases atherosclerosis and obesity through enhanced type I interferon signaling. Cell Metab. (2014) 20:813–26. 10.1016/j.cmet.2014.09.016 25440058 PMC4254584

[9] Sena LA Chandel NS. Physiological roles of mitochondrial reactive oxygen species. Mol Cell. (2012) 48:158–67. 10.1016/j.molcel.2012.09.025 23102266 PMC3484374

[10] Portela A Esteller M. Epigenetic modifications and human disease. Nat Biotechnol. (2010) 28:1057–68. 10.1038/nbt.1685 20944598

